# Supplementary material for: Vulnerability of drug‐resistant EML4‐ALK rearranged lung cancer to transcriptional inhibition
Source: EMBO Mol Med. 2020 Jun 17;12(7):e11099. doi: 10.15252/emmm.201911099 (PMC7338803; doi:10.15252/emmm.201911099)
Supplement: Supplementary file 1 — Appendix [file EMMM-12-e11099-s001.pdf]

Vulnerability of drug-resistant EML4-ALK rearranged lung cancer to transcriptional inhibition

Athanasios R. Paliouras[1,2‡], Marta Buzzetti [1,3‡], Lei Shi[1,2‡], Ian Donaldson[4], Peter Magee[1,2], Sudhakar Sahoo[5], Hui-Sun Leong[5], Matteo Fassan[6], Matthew Carter[2,7], Gianpiero Di Leva[8], Matthew Krebs[2,7], Fiona Blackhall[2,7], Christine M. Lovly[9] and Michela Garofalo[1,2 *]

Appendix-Table of contents

-Appendix Figure S1

-Appendix Figure S2

-Appendix Figure S3

-Appendix Figure S4

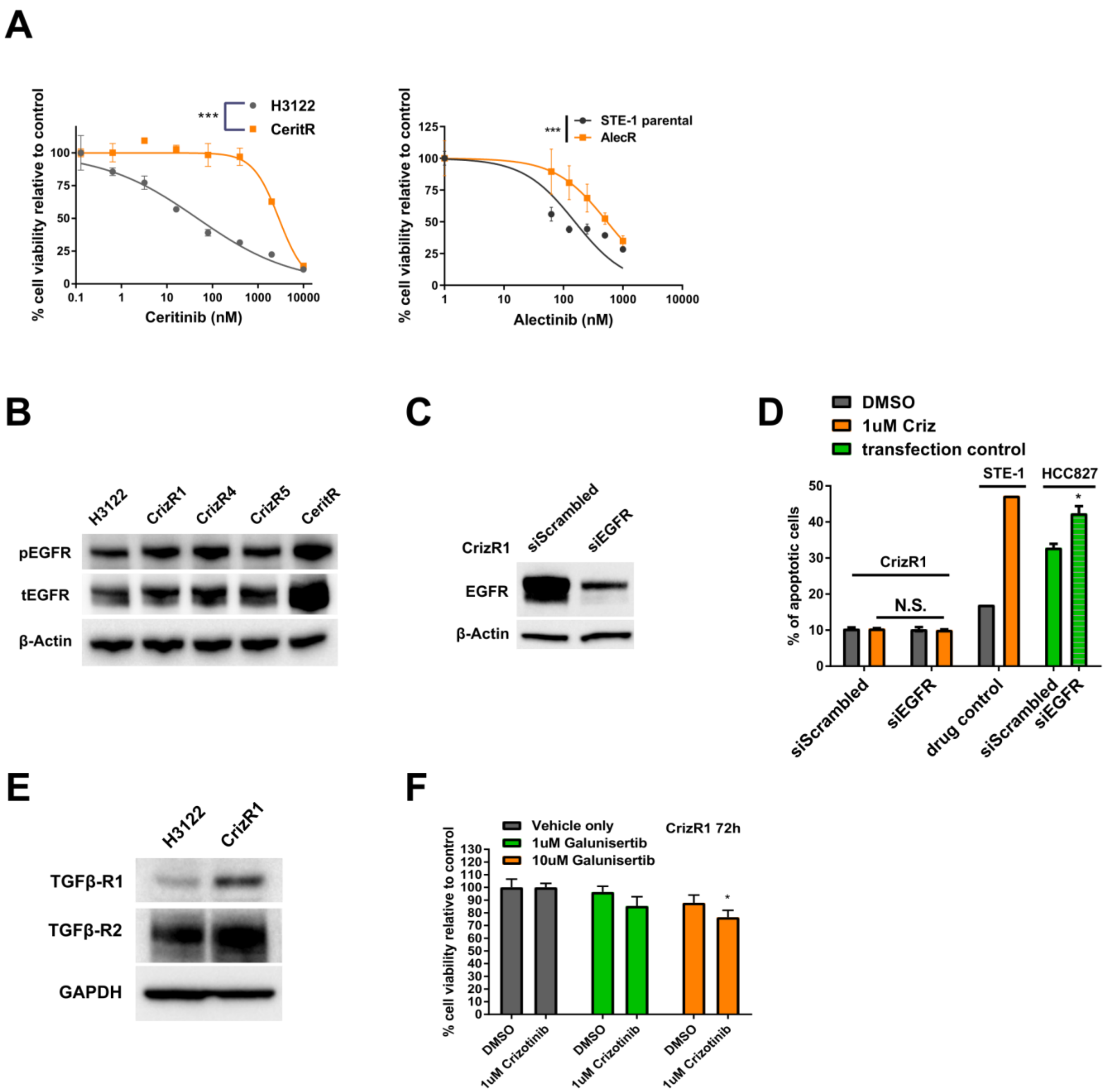

Appendix Fig S1

Appendix Figure S1: EGFR or TGFβ-R activation is not enough to account for crizotinib resistance. A) Cell viability assay of ceritinib and alectinib resistant cells compared to parental isogenic cell lines (n=4). **B)** Western blot analysis of the indicated proteins in H3122 parental and isogenic drug-resistant cell lines. **C)** CrizR1 cells were transfected with siScrambled or siEGFR and cell extracts analysed by western blotting. **D)** CrizR1 and HCC827 ($EGFR_{mut}$) cells were transfected with siScrambled or siEGFR, treated with DMSO or 1uM crizotinib for 48h, stained with Annexin V/PI and analysed using flow cytometry for Annexin V+ cells. STE-1 cells were treated with DMSO or 1uM crizotinib as drug control (n=2). **E)** Western blot analysis for TGFβ-R1 and TGFβ-R2 in H3122 parental and isogenic crizotinib-resistant cells. **F)** Proliferation assay of CrizR1 cells treated with 1uM or 10uM of galunisertib ± 1uM crizotinib for 48h (n=4).

Plotted graphs show mean ± SD. Statistical comparisons were performed using a paired, two-tailed student's t-test. *$P < 0.05$, ***$P < 0.001$, N.S. = Not Significant $P > 0.05$.

A

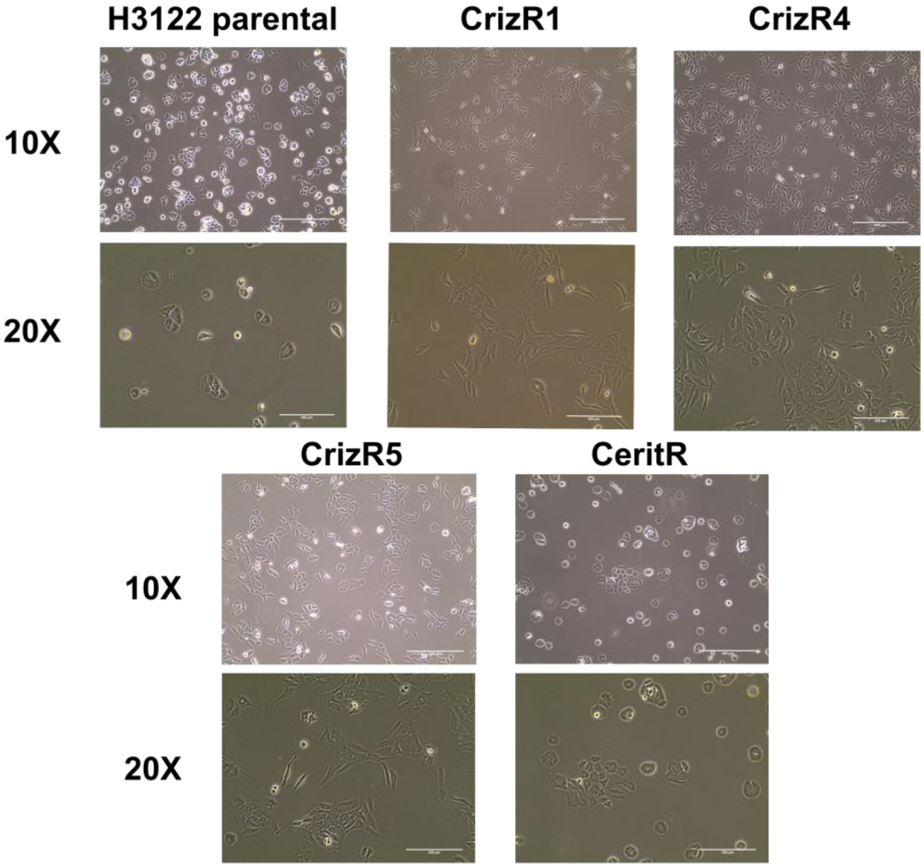

B

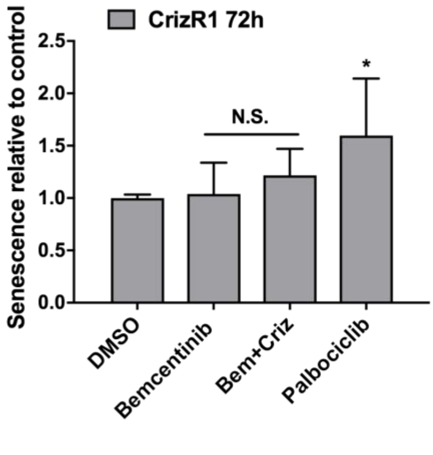

Appendix Fig S2

Appendix Figure S2: Crizotinib resistant cells exhibit a mesenchymal-like morphology. A) Representative pictures of H3122 sensitive, CrizR1 and CrizR4 resistant cells at 10X (scale bars=400um) and 20X (scale bars=200um) magnification. **B)** Senescence assay of CrizR1 cells after 72h treatment with bemcentinib (2.5uM), bemcentinib+crizotinib (2.5uM and 1uM, respectively) and palbociclib (5uM) (n=2). Plotted graph shows mean ± SD. Statistical comparisons were performed using a paired, two-tailed student's t-test. *$P < 0.05$, N.S. = Not Significant $P > 0.05$.

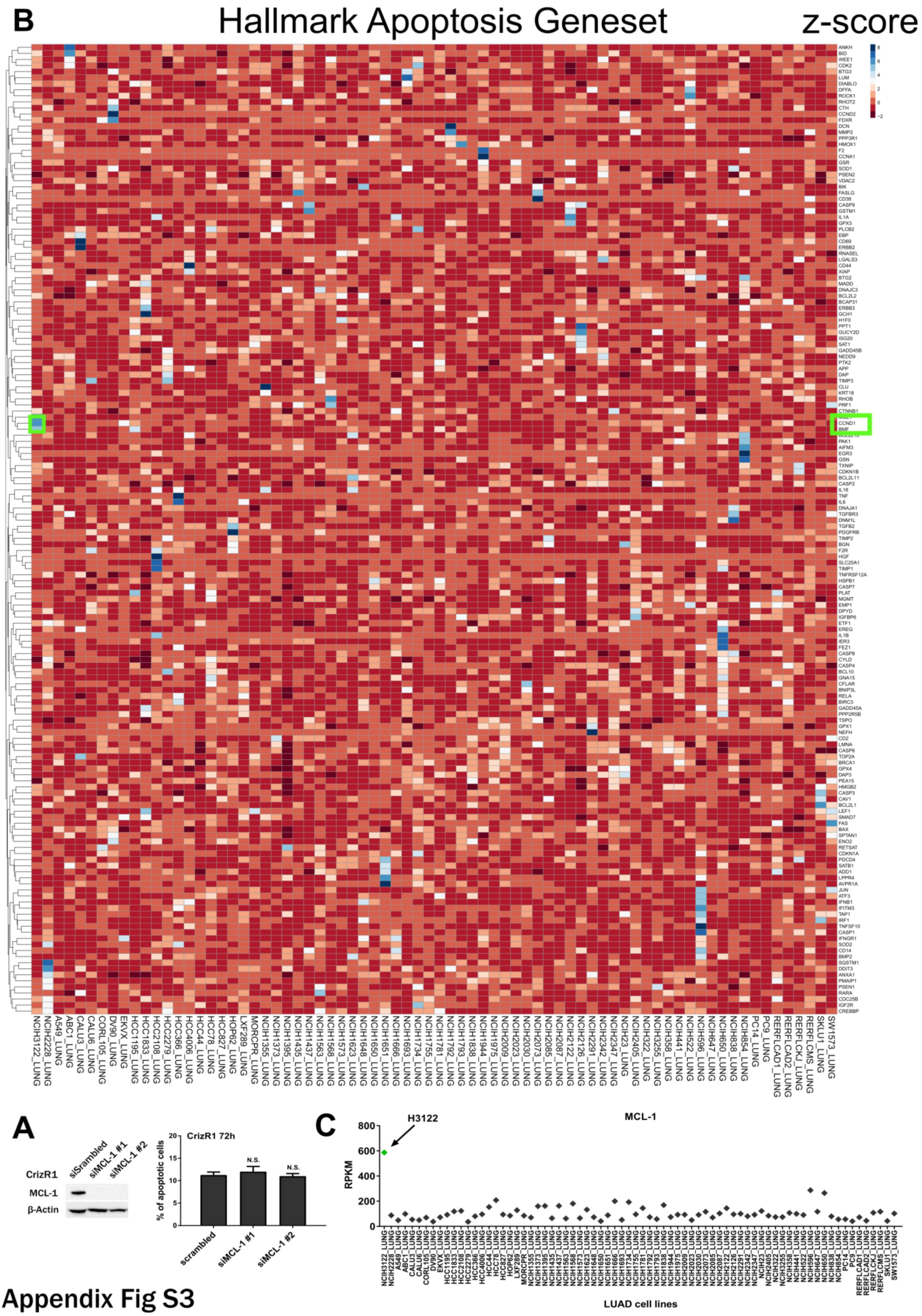

Appendix Fig S3

Appendix Figure S3: H3122 EML4-ALK cells have exceptionally high expression levels of MCL-1 and CCND1 compared to other LUAD cells. A) (Left) Western blot analysis of CrizR1 cells treated with siScrambled or siRNA for MCL-1 for 72h. (Right) CrizR1 were treated as above, for 72h and cells were stained with Annexin V/PI and analysed by flow cytometry for Annexin V+ cells 72h post-transfection (n=3). Plotted graph shows mean ± SD. Statistical comparisons were performed using a paired, two-tailed student's t-test. N.S. = Not Significant $P > 0.05$. **B)** Heatmap plotting the z-score of all the apoptosis-related genes from CCLE RNA-seq data comparing the LUAD cell lines. Highlighted with green are the 2 most upregulated antiapoptotic genes in H3122 cells. **C)** The same dataset as B) was used, and RPKM values were plotted for the *MCL-1* gene, indicating high expression in H3122 cells compared with other LUAD cells.

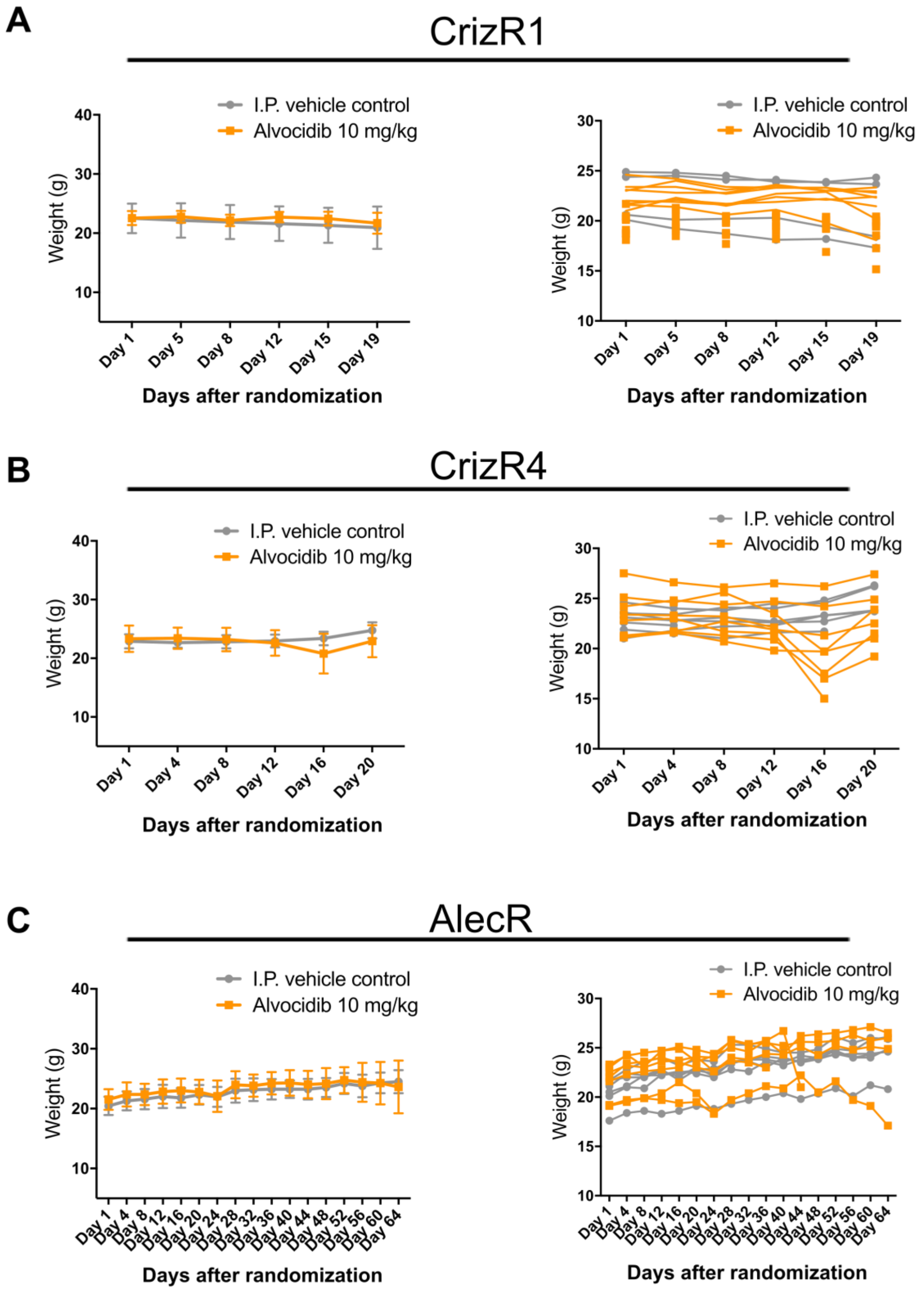

Appendix Fig S4

Appendix Figure S4: Body weight of mice treated with alvocidib or placebo.

A-C) Average body weight of all mice (left) and body weight of each single mouse (right) injected with CrizR1 cell lines in response to either vehicle control or alvocidib (Control n=5; alvocidib n=8). **B)** Average body weight of all mice (left) and body weight of each single mouse (right) injected with CrizR4 cells in response to either vehicle control or alvocidib (Control n=8; alvocidib n=8). **C)** Average body weight of all mice (left) and body weight of each single mouse (right) injected with AlectR cell lines in response to either vehicle control or alvocidib (Control n=7; alvocidib n=7). Plotted graphs on the left show mean ± SD.
