# Supplementary material for: Vulnerability of drug‐resistant EML4‐ALK rearranged lung cancer to transcriptional inhibition
Source: EMBO Mol Med. 2020 Jun 17;12(7):e11099. doi: 10.15252/emmm.201911099 (PMC7338803; doi:10.15252/emmm.201911099)
Supplement: Supplementary file 2 — Expanded View Figures PDF [file EMMM-12-e11099-s002.pdf]

# Expanded View Figures

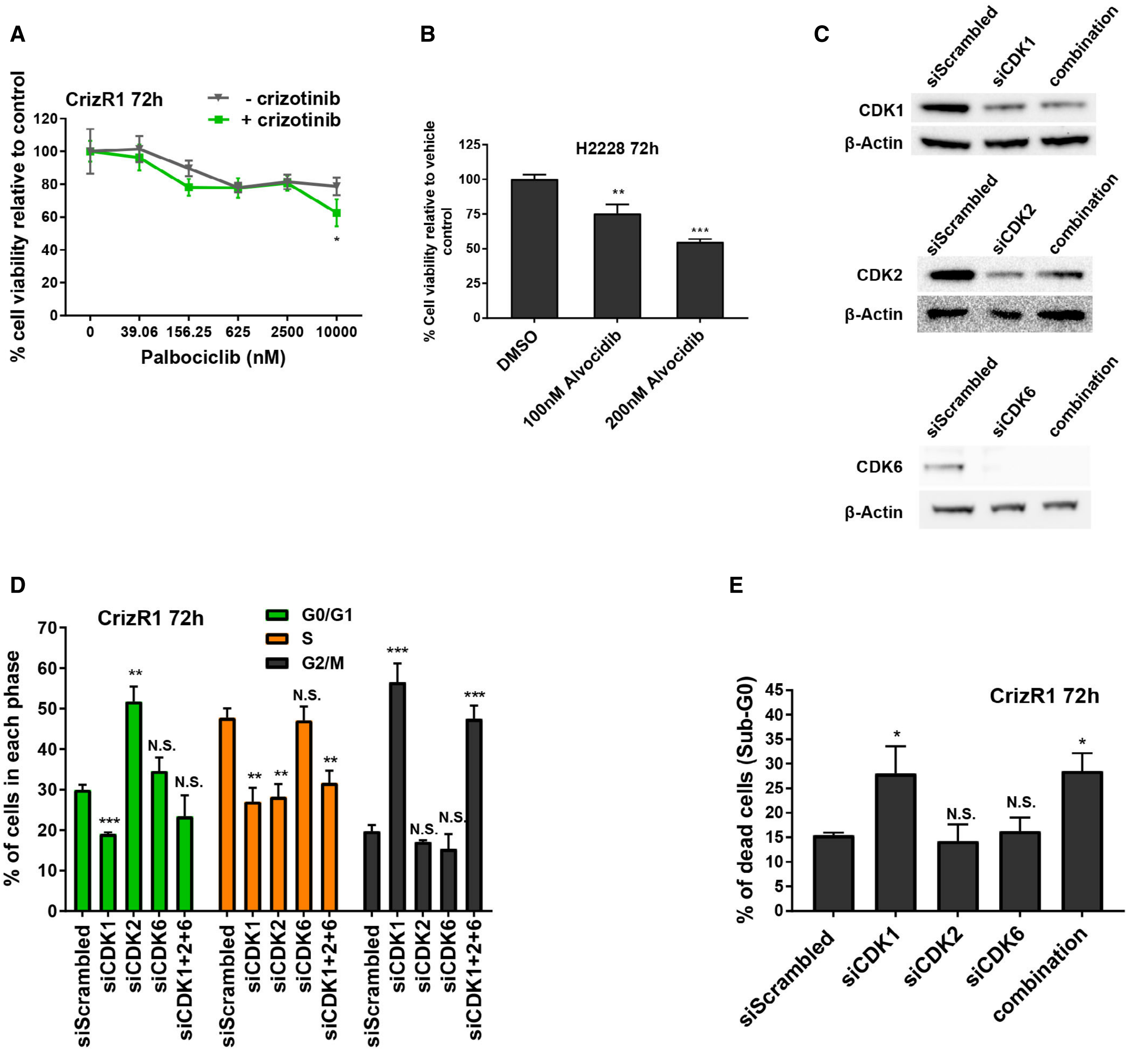


**Figure EV1.**

**Figure EV1. CDK6 upregulation does not account for crizotinib resistance.**

A Cell viability assay of CrizR1 cells treated with the indicated concentrations of palbociclib ± 1 μM crizotinib for 72 h. H3122 cells were treated in parallel with DMSO/1 μM crizotinib as drug control ($n$ = 4). *$P$ = 0.02.

B Proliferation assay of H2228 cells treated with the indicated concentrations of alvocidib or vehicle control for 72 h ($n$ = 4). 100 nM alvocidib, $P$ = 0.002; 200 nM alvocidib, $P$ = 0.0001.

C Western blot analysis for the indicated proteins after transfection of CrizR1 cells with CDK1, CDK2 and CDK6 siRNAs.

D, E Cell cycle analysis (D) and sub-G0 (cell death) quantification (E) of crizotinib-resistant cells transfected with the indicated siRNAs, fixed and stained with propidium iodide and analysed with flow cytometry after 72 h. D) Phase G0/G1 siCDK1 $P$ = 0.001, siCDK2 $P$ = 0.009, siCDK6 $P$ = 0.2, combination $P$ = 0.1; Phase S siCDK1 $P$ = 0.008; siCDK2 $P$ = 0.004, siCDK6 $P$ = 0.8, combination $P$ = 0.009; Phase G2/M siCDK1 $P$ = 0.003, siCDK2 $P$ = 0.1, siCDK6 $P$ = 0.1, combination $P$ = 0.002 E) siCDK1 versus siControl $P$ = 0.02, siCDK2 versus siControl $P$ = 0.59; siCDK6 versus siControl$P$ = 0.59, combination versus siControl $P$ = 0.03.

Data information: Statistical comparisons were performed using a paired, two-tailed Student $t$-test. Plotted graphs show mean ± SD ($n$ = 3, unless otherwise specified). *$P < 0.05$, **$P < 0.01$, ***$P < 0.001$, N.S. = Not Significant $P > 0.05$.
Source data are available online for this figure.

 © 2020 The Authors

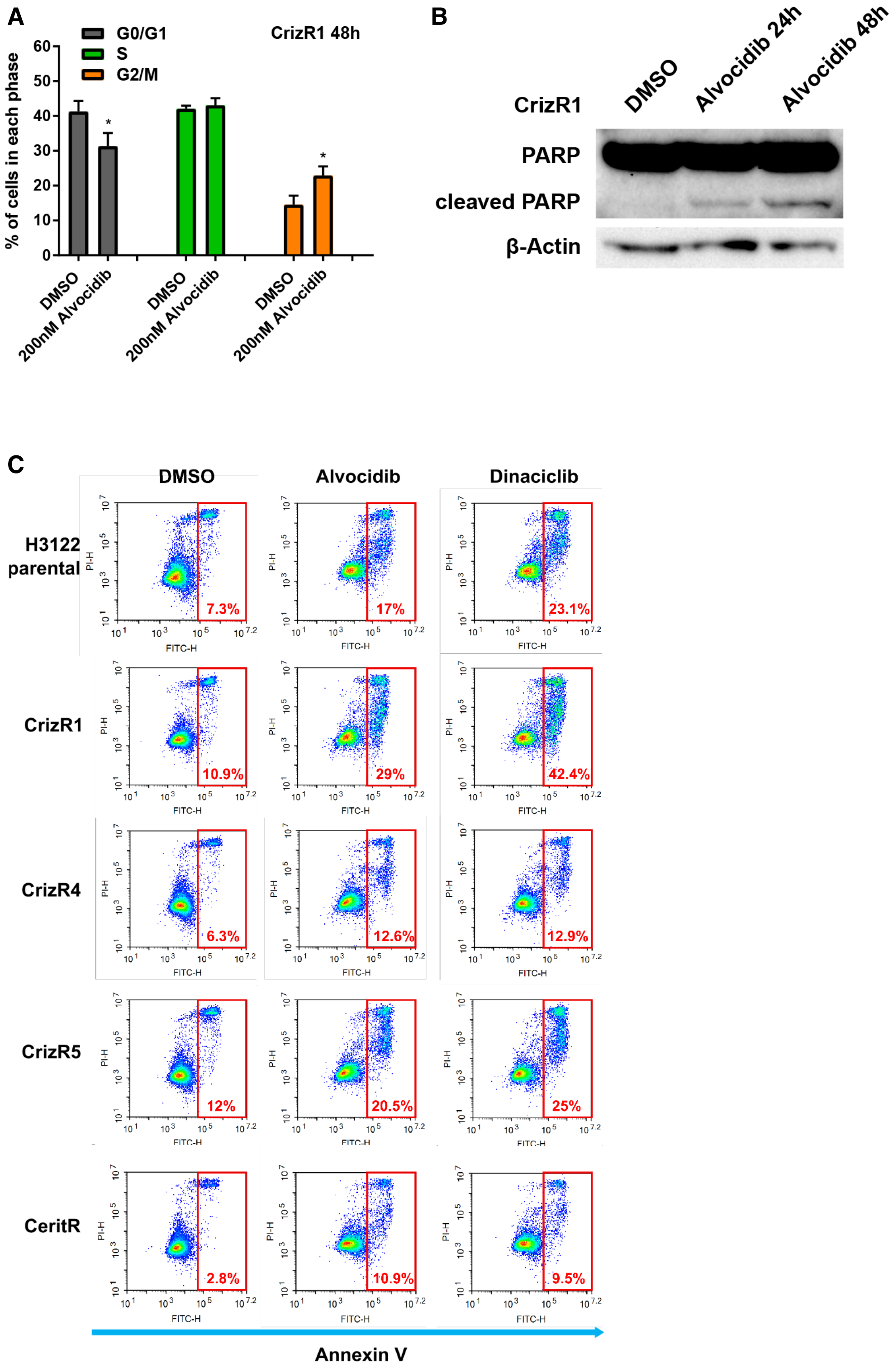
A
G0/G1
S
G2/M
CrizR1 48h
% of cells in each phase
DMSO
200nM Alvocidib
B
CrizR1
DMSO
Alvocidib 24h
Alvocidib 48h
PARP
cleaved PARP
β-Actin
C
DMSO
Alvocidib
Dinaciclib
H3122 parental
CrizR1
CrizR4
CrizR5
CeritR
7.3%
17%
23.1%
10.9%
29%
42.4%
6.3%
12.6%
12.9%
12%
20.5%
25%
2.8%
10.9%
9.5%
PI-H
FITC-H
Annexin V

**Figure EV2.**

**Figure EV2. CDK inhibition has a more potent apoptotic effect compared with cell cycle arrest.**

A Cell cycle analysis of CrizR1 cells treated with DMSO or 200 nM alvocidib for 24 h. G0/G1 alvocidib versus DMSO $P = 0.02$; S alvocidib versus DMSO $P = 0.5$; G2/M alvocidib versus DMSO $P = 0.02$.
B PARP protein cleavage of CrizR1 cells treated with DMSO or 200 nM alvocidib and analysed by Western blotting.
C Apoptotic assay in the indicated cell lines after treatment with the indicated compounds for 48 h. Cells were stained with Annexin V/PI and Annexin V+ cells were analysed and quantified by flow cytometry. Representative plots of 2 biological replicates are shown.

Data information: Statistical comparisons were performed using a paired, two-tailed Student *t*-test. Plotted graphs show mean ± SD ($n = 3$, unless otherwise specified). $^*P < 0.05$.
Source data are available online for this figure.

**Figure EV3. Alvocidib elicits EML4-ALK NSCLC cell-specific apoptosis.**

A Apoptotic assay in CrizR1 and HBEC cells treated with 200 nM alvocidib for 48 h. Cells were stained with Annexin V/PI and Annexin V+ cells were analysed and quantified by flow cytometry. Representative plots of 3 biological replicates are shown. CrizR1 alvocidib versus DMSO $P = 0.001$; HBEC alvocidib versus DMSO $P = 0.3$.
B Apoptotic assay for the indicated cell lines treated with DMSO, 200 nM alvocidib or 25 nM dinaciclib for 48 h. Cells were stained with Annexin V/PI and Annexin V+ cells were analysed and quantified by flow cytometry. Representative plots of 3 biological replicates are shown.
C CrizR1 cells were treated with DMSO or 200 nM alvocidib for 24 h. Protein extracts were hybridized to a 43-antibody array and analysed by immunoblotting. Graphs depict all the means ± SD ($n = 2$). BAD $P = 0.015$; BID $P = 0.001$; BIM $P = 0.01$; Caspase 3 $P = 0.04$; Caspase 8 $P = 0.01$; DR6 $P = 0.011$; FasL $P = 0.02$; HSP60 $P = 0.001$; HSP70 $P = 0.02$; HTRA $P = 0.002$; IGF-I $P = 0.02$; IGF-II $P = 0.004$; IGFBP-2 $P = 0.003$; IGFBP-3 $P = 0.04$; IGFBP-5 $P = 0.03$; Livin $P = 0.03$; p27 $P = 0.04$; SMAC $P = 0.01$; TrailR-3 $P = 0.02$.
D RNA Polymerase II phosphorylation at Ser2 by Western blot analysis. CrizR1 cells were treated with 200 nM alvocidib, 25 nM dinaciclib or DMSO for 6 h.
E CrizR1 cells were transfected with siRNA Control (Scrambled) and CDK7 or CDK9 siRNAs for 72 h or treated with DMSO/200 nM alvocidib/50 nM THZ1. Cells were stained with Annexin V/PI and analysed by flow cytometry for Annexin V+ cells 72 h post-transfection.

Data information: Statistical comparisons were performed using a paired, two-tailed Student *t*-test. Plotted graphs show mean ± SD ($n = 3$, unless otherwise specified). $^*P < 0.05$, $^{***}P < 0.001$, N.S. = Not Significant $P > 0.05$.
Source data are available online for this figure.

 © 2020 The Authors

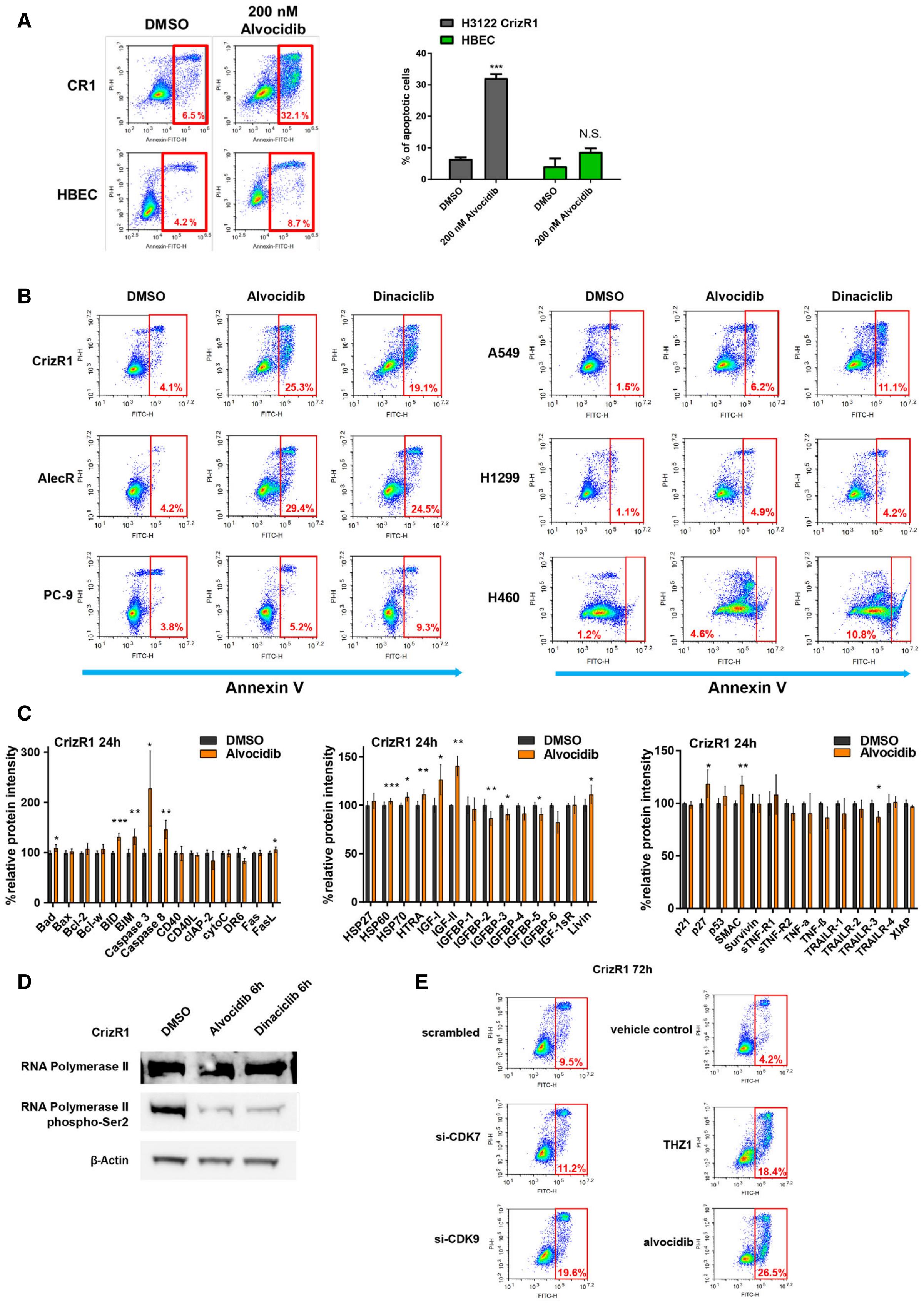
A
DMSO
200 nM Alvocidib
CR1
HBEC
6.5 %
32.1 %
4.2 %
8.7 %
H3122 CrizR1
HBEC
% of apoptotic cells
N.S.
DMSO
200 nM Alvocidib
B
DMSO
Alvocidib
Dinaciclib
CrizR1
AlecR
PC-9
A549
H1299
H460
4.1%
25.3%
19.1%
4.2%
29.4%
24.5%
3.8%
5.2%
9.3%
1.5%
6.2%
11.1%
1.1%
4.9%
4.2%
1.2%
4.6%
10.8%
Annexin V
C
CrizR1 24h
DMSO
Alvocidib
%relative protein intensity
Bad
Bax
Bcl-2
Bcl-w
BID
BIM
Caspase 3
Caspase 8
CD40
CD40L
cIAP-2
cytoC
DR6
Fas
FasL
HSP27
HSP60
HSP70
HTRA
IGF-I
IGF-II
IGFBP-1
IGFBP-2
IGFBP-3
IGFBP-4
IGFBP-5
IGFBP-6
IGF-1sR
Livin
p21
p27
p53
SMAC
Survivin
sTNF-R1
sTNF-R2
TNF-a
TNF-ß
TRAILR-1
TRAILR-2
TRAILR-3
TRAILR-4
XIAP
D
CrizR1
DMSO
Alvocidib 6h
Dinaciclib 6h
RNA Polymerase II
RNA Polymerase II phospho-Ser2
β-Actin
E
CrizR1 72h
scrambled
si-CDK7
si-CDK9
vehicle control
THZ1
alvocidib
9.5%
11.2%
19.6%
4.2%
18.4%
26.5%

**Figure EV3.**

**Figure EV4. Alvocidib is effective *in vivo* in crizotinib-resistant tumours.**

A Characterization of drug-resistant cell lines: CrizR1, CrizR4 and AlecR cells were kept in absence of crizotinib or alectinib for up to 6 weeks (−drug). Cell viability was assessed by MTS assay in the presence of 1 μM crizotinib or 500 nM alectinib and compared with viability of cells maintained in culture with the drugs (+ drug). Parental sensitive cells were used as positive control. Plotted graph show mean ± SD ($n$ = 5). ***$P < 0.0001$.

B Tumour growth of *in vivo* xenografts of H3122 parental cell lines in response to crizotinib or alvocidib and correspondent tumour weights (P.O. control $n$ = 5; I.P control $n$ = 5; crizotinib $n$ = 6 and alvocidib $n$ = 4). Tumour weights, crizotinib $P$ = 0.002; alvocidib $P$ = 0.002.

C Photograph of H3122 tumours treated with vehicle, crizotinib or alvocidib.

D Photographs of CrizR1 tumours treated with vehicle, crizotinib or alvocidib.

E Tumour growth of *in vivo* xenografts of CrizR4 cells. $2.5 \times 10^6$ cells were injected subcutaneously, and mice were treated daily by oral gavage with either vehicle control, crizotinib or alvocidib (I.P. control $n$ = 5; alvocidib $n$ = 7).

F, G Photographs of (F) CrizR4 and (G) AlecR tumours treated with vehicle or alvocidib.

Data information: Statistical comparisons were performed using a paired, two-tailed Student $t$-test. Plotted graphs show mean ± SD. *$P < 0.05$, **$P < 0.01$, ***$P < 0.001$, N.S. = Not Significant $P > 0.05$.


© 2020 The Authors

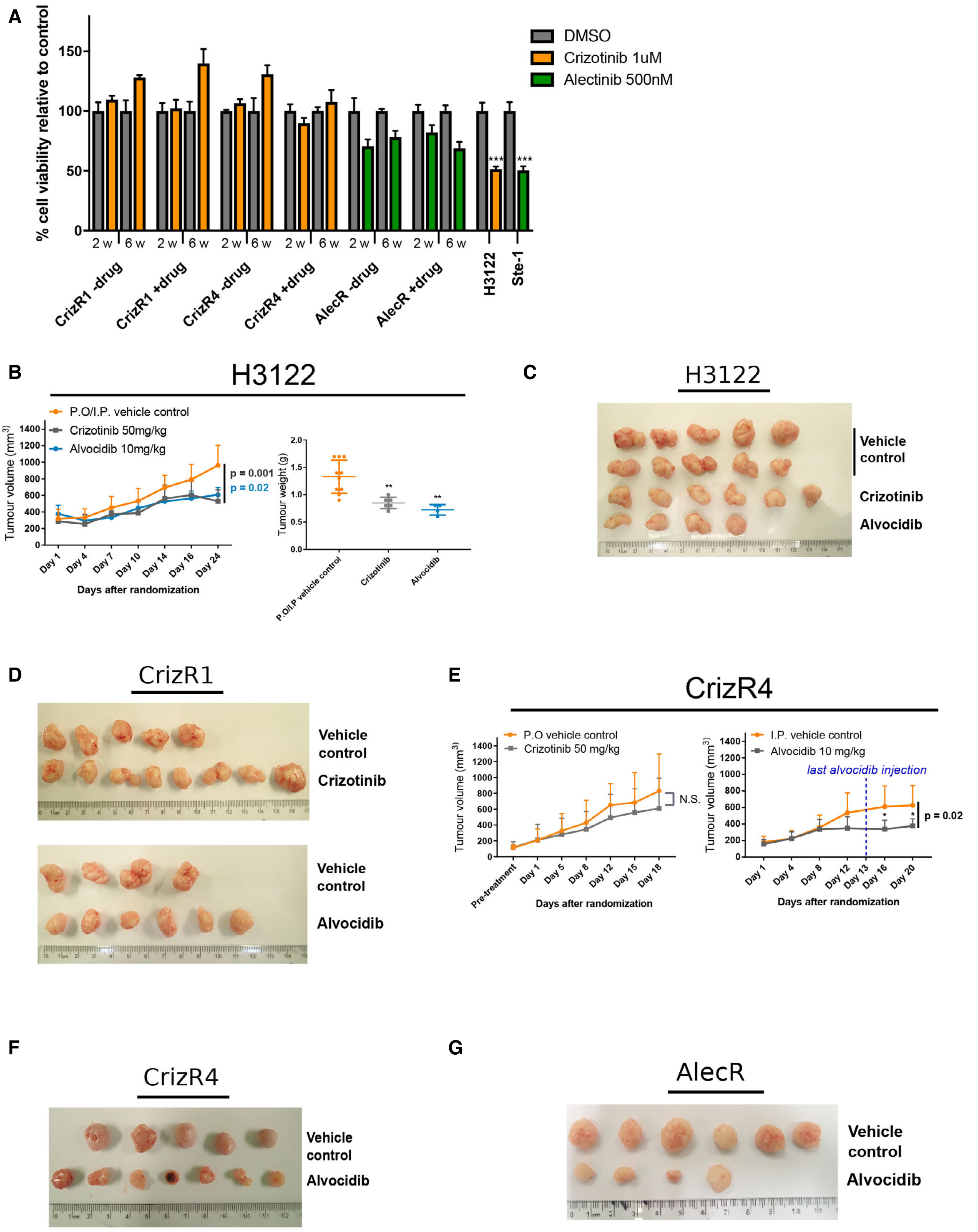
A
% cell viability relative to control
DMSO
Crizotinib 1uM
Alectinib 500nM
CrizR1 -drug
CrizR1 +drug
CrizR4 -drug
CrizR4 +drug
AlecR -drug
AlecR +drug
H3122
Ste-1
B
H3122
P.O/I.P. vehicle control
Crizotinib 50mg/kg
Alvocidib 10mg/kg
Tumour volume (mm3)
p = 0.001
p = 0.02
Days after randomization
Tumour weight (g)
P.O/I.P vehicle control
Crizotinib
Alvocidib
C
H3122
Vehicle control
Crizotinib
Alvocidib
D
CrizR1
Vehicle control
Crizotinib
Vehicle control
Alvocidib
E
CrizR4
P.O vehicle control
Crizotinib 50 mg/kg
N.S.
I.P. vehicle control
Alvocidib 10 mg/kg
last alvocidib injection
p = 0.02
Days after randomization
F
CrizR4
Vehicle control
Alvocidib
G
AlecR
Vehicle control
Alvocidib

**Figure EV4.**

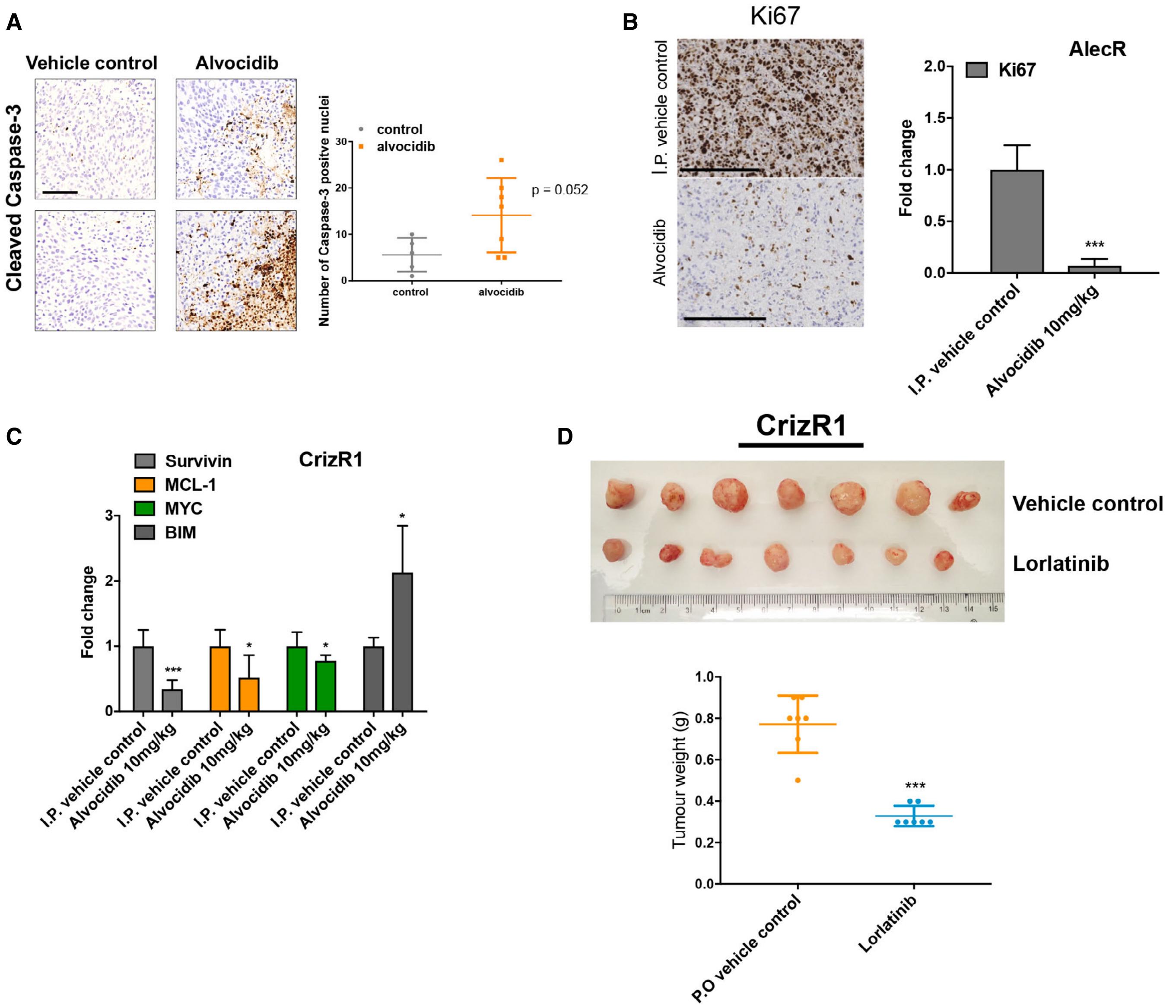


**Figure EV5. Alvocidib reduces tumour growth *in vivo* via MYC downregulation and BIM upregulation.**

A IHC for cleaved caspase 3 and relative quantification from CrizR4 xenograft mouse model. Scale bar = 100 μm.
B Ki67 immunohistochemical (IHC) staining in AlectR xenograft tumours. Scale bar = 200 μm. Quantitative analysis of Ki67 staining is reported on the right. $P = 0.0004$.
C qPCR for *Survivin*, *MCL-1*, *MYC* and *BIM* in CrizR1 xenografts. Survivin $P = 0.0003$; MCL-1 $P = 0.04$; MYC $P = 0.03$; BIM $P = 0.014$.
D Photograph and weights of CrizR1 tumours treated with vehicle or lorlatinib. Tumour weights $P < 0.0001$.

Data information: Statistical comparisons were performed using a paired, two-tailed Student *t*-test. Plotted graphs show mean ± SD. *$P < 0.05$, ***$P < 0.001$, N.S. = Not Significant $P > 0.05$.


© 2020 The Authors
