# Supplementary figures and images for: Vulnerability of drug‐resistant EML4‐ALK rearranged lung cancer to transcriptional inhibition
Source: EMBO Mol Med. 2020 Jun 17;12(7):e11099. doi: 10.15252/emmm.201911099 (PMC7338803; doi:10.15252/emmm.201911099)

1E

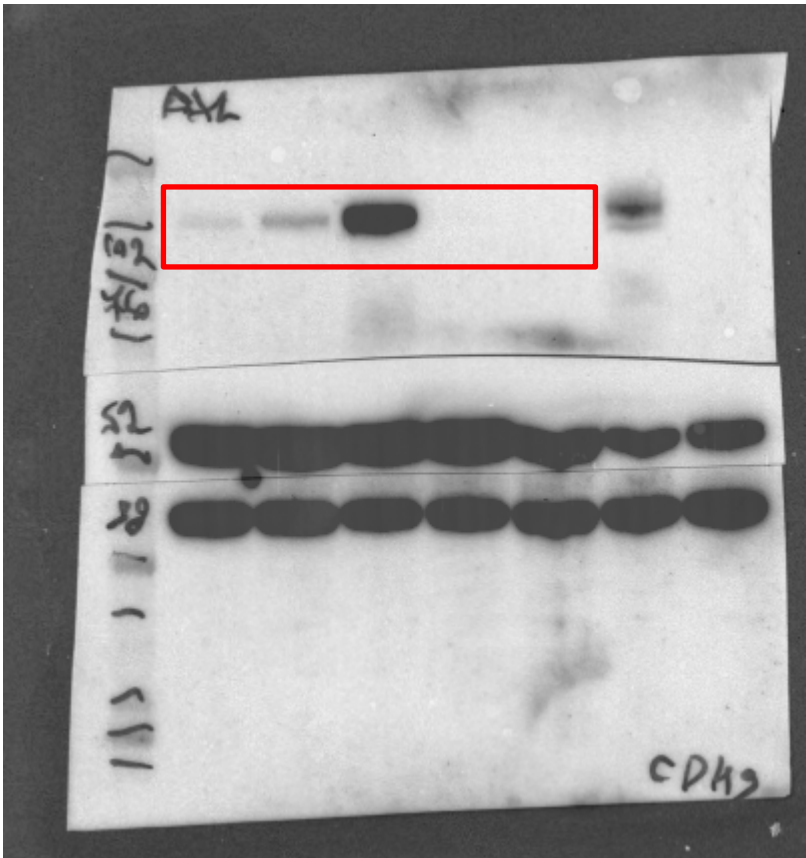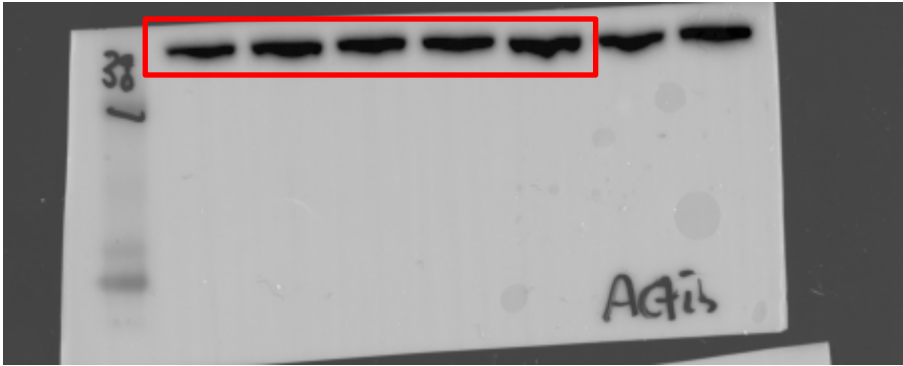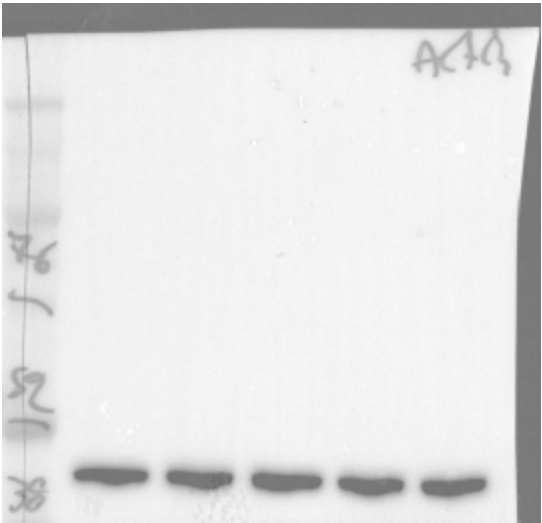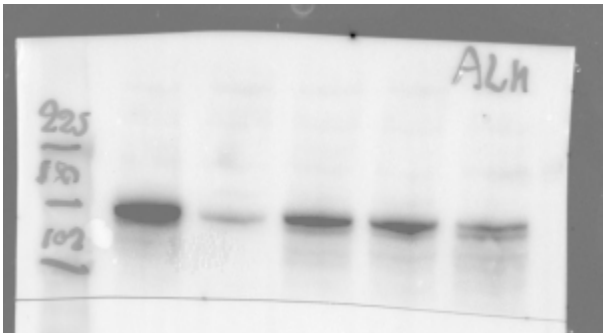

Supplement: Supplementary file 8 — Source Data for Figure 1 [file EMMM-12-e11099-s006.pdf]

2C

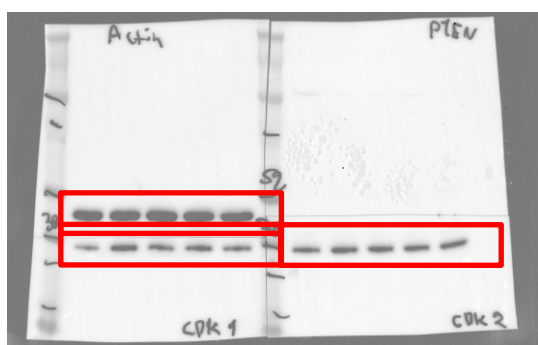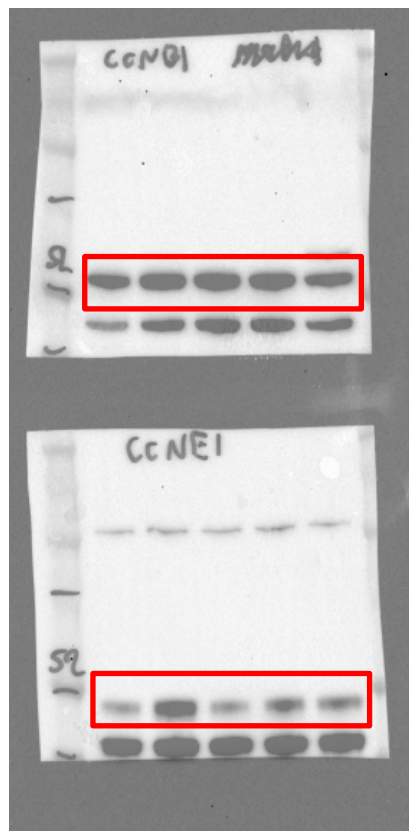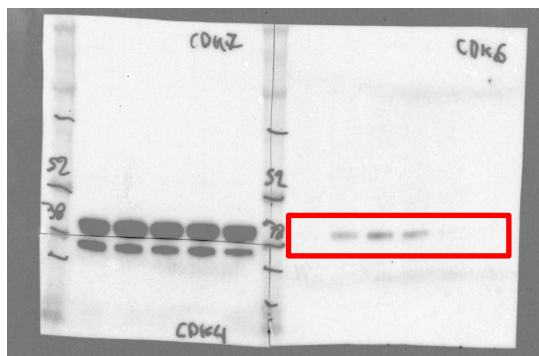

2D

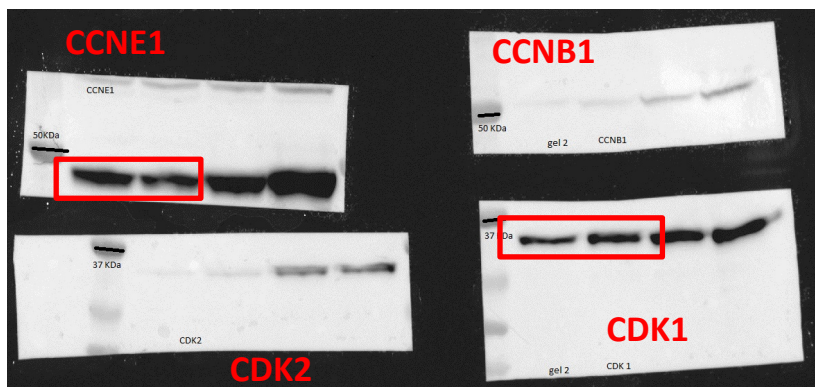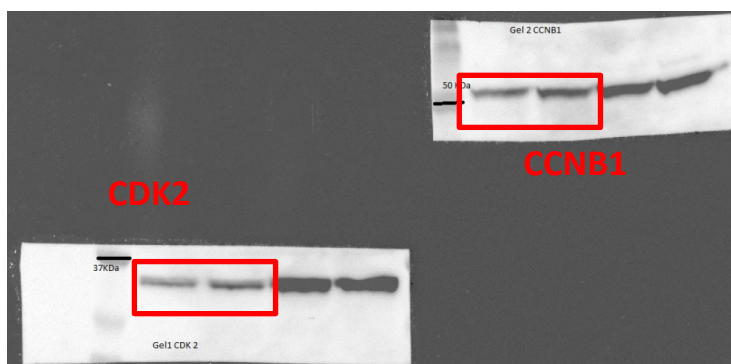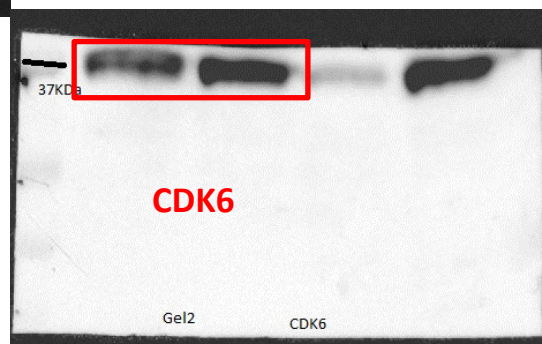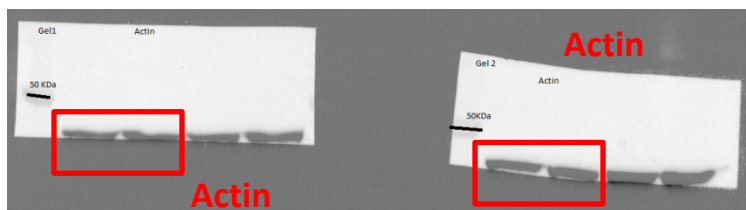

Supplement: Supplementary file 9 — Source Data for Figure 2 [file EMMM-12-e11099-s007.pdf]

4C

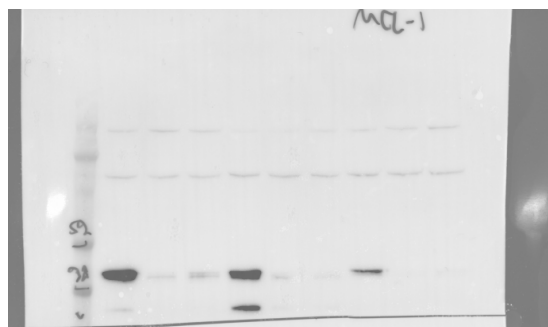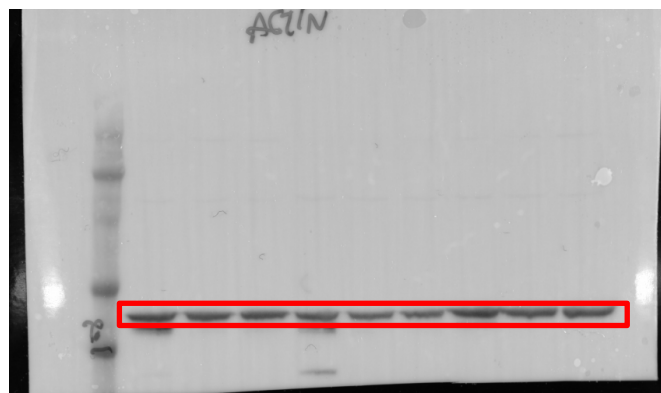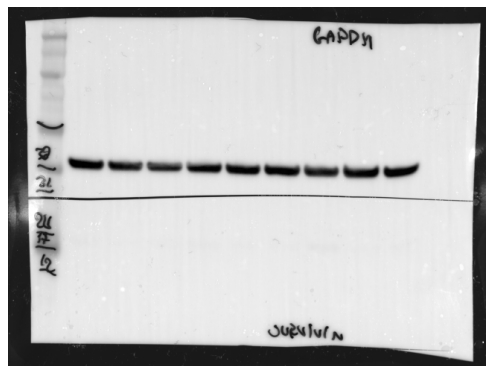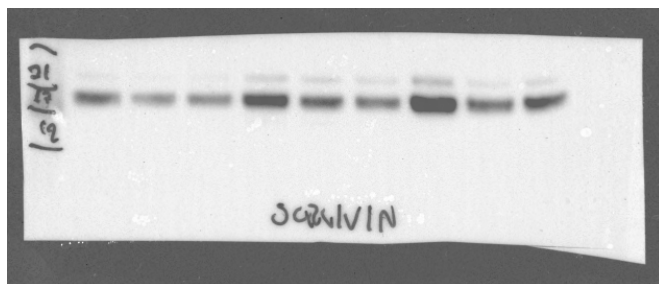

4D

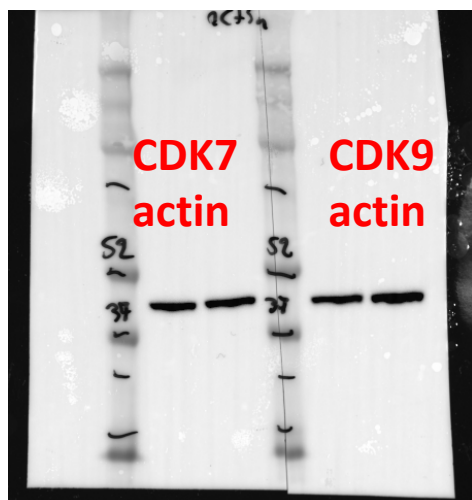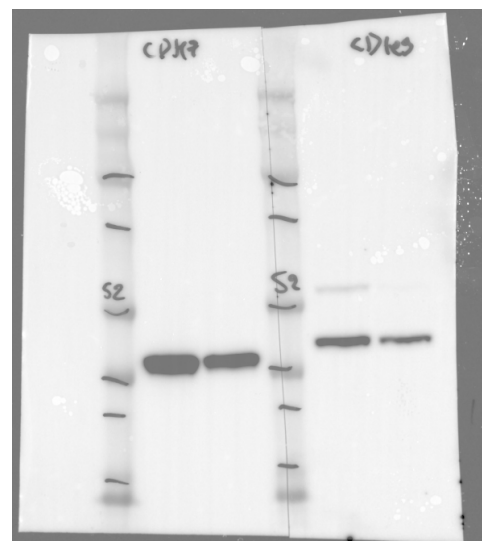

4G

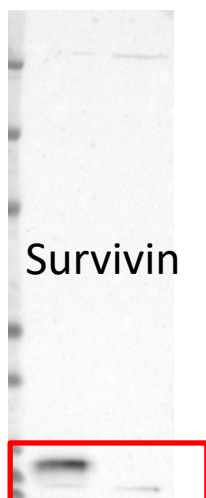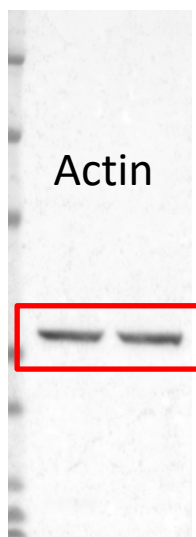

Supplement: Supplementary file 10 — Source Data for Figure 4 [file EMMM-12-e11099-s008.pdf]

5F

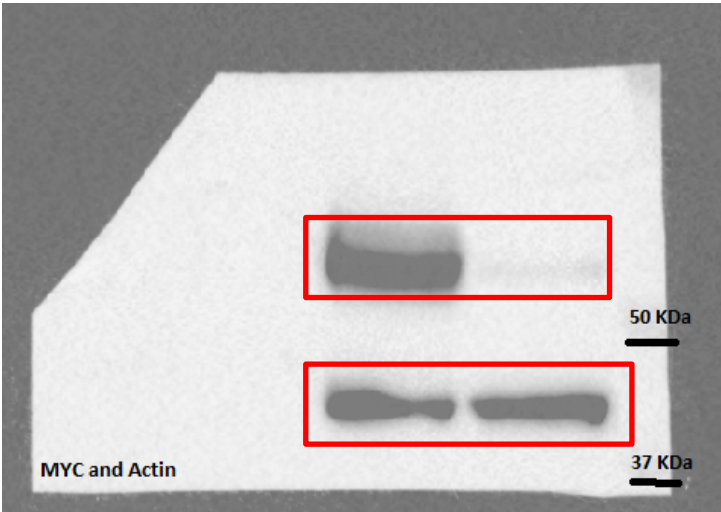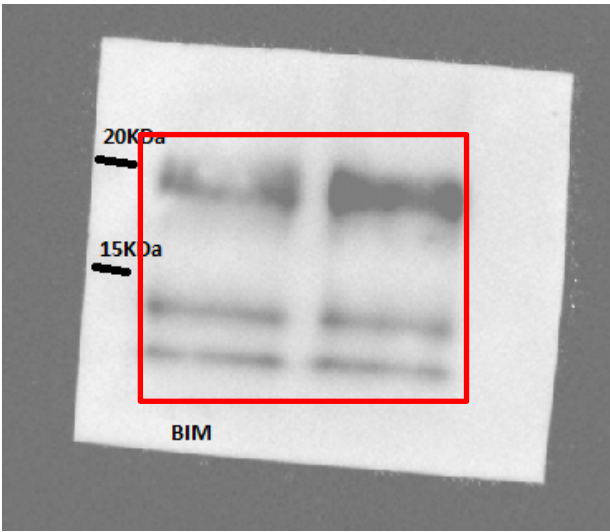

Supplement: Supplementary file 11 — Source Data for Figure 5 [file EMMM-12-e11099-s009.pdf]
