# Supplementary material for: Vulnerability of drug‐resistant EML4‐ALK rearranged lung cancer to transcriptional inhibition
Source: EMBO Mol Med. 2020 Jun 17;12(7):e11099. doi: 10.15252/emmm.201911099 (PMC7338803; doi:10.15252/emmm.201911099)

EV1C

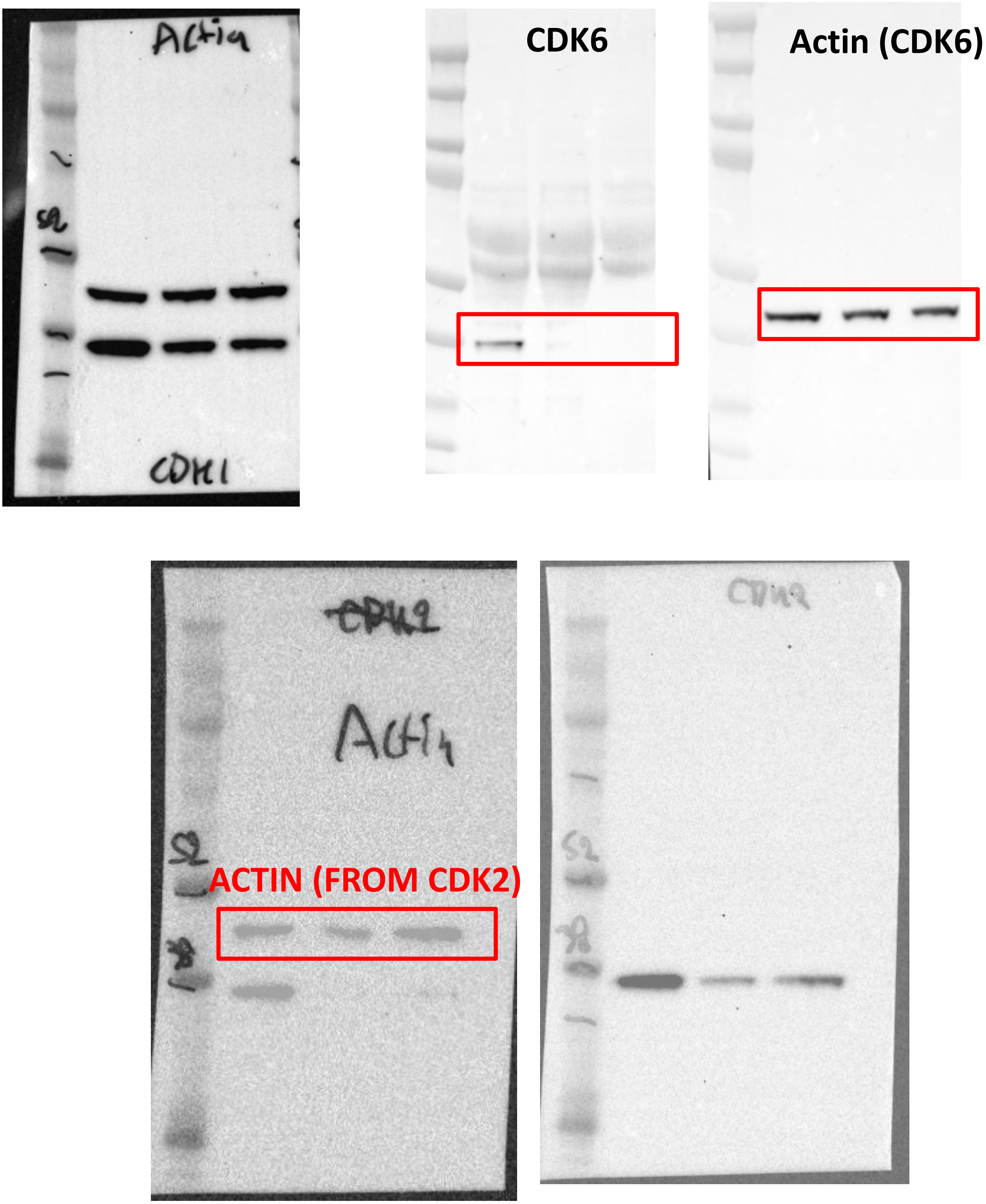

Supplement: Supplementary file 6 — Source Data for Expanded View and Appendix [file EMMM-12-e11099-s010.zip › Source Data for EV Figures.zip!/Source_data_for_EV1.pdf]
